# Influence of glutathione s-transferase on the ototoxicity caused by aminoglycosides

**DOI:** 10.1590/S1808-86942010000300006

**Published:** 2015-10-20

**Authors:** Bruna Palodetto, Mariana Postal, Carlos Roberto Escrivão Grignoli, Edi Lúcia Sartorato, Camila Andréa de Oliveira

**Affiliations:** 1 Undergraduate student – Biomedics; 2 Undergraduate student, Biomedics; 3 MSc. Adjunct Professor – Herminio Ometto University – UNIARARAS; 4 Associate Professor; Researcher – Molecular Biology and Genetic Engineering Center – CBMEG – UNICAMP; 5 PhD; Assistant Professor – Health Sciences Center – NUCISA – Hermínio Ometto University – UNIARARAS

**Keywords:** aminoglycosides, molecular biology, oxidative stress, hearing loss, genetic polymorphism

## Abstract

The process of hair cell damage and death as a result of exposure to noise and ototoxins seems to be mediated by reactive oxygen species.

**Aim:** To investigate the relationship between genetic polymorphisms in the Glutathione S-transferase and the susceptibility to hearing loss induced by aminoglycosides.

**Materials and Methods:** Null genotypes were analyzed by multiplex-PCR in the DNA samples from 50 patients and 72 controls. The patients were divided into 3 groups, 10 with hearing loss using aminoglycosides (group A), 20 with hearing loss without exposure to the drug (group B) and 20 hearing individuals who used the antibiotic (group C).

**Study Design:** Experimental.

**Results:** Polymorphisms in the GSTM1 and GSTT1 genes were found in 16% and 42% of patients and in 18% and 53% of the control group, respectively. After statistical analysis no significant difference was observed between the control groups and A (p=0.86) and (p=0.41), controls and B (p=0.27) and (p=0.24), controls and C (p=0.07) and (p=0.47), controls and A + C (p=0.09) and (p=0.47), C and A (p=0.32) and (p=0.75), GSTT1 and GSTM1, respectively.

**Conclusion:** Our data show that polymorphisms in GSTM1 and GSTT1 genes have no influence on the ototoxicity of aminoglycosides.

## INTRODUCTION

Many are the factors responsible for hearing impairment (HI), and they include the environment, infections, noise and ototoxic agents – which damage the auditory nerve; or genetics^1^. Among the genetic cases, it is believed that there are more than 100 genes involved in the etiology of non-syndromic sensorineural hearing loss^2^.

Oxygen reactive species (ORS) are involved in a growing number of diseases, including hearing loss – in a lesser frequency. Nonetheless, oxidative stress is strongly related to ototoxic-induced hearing deficiency caused by aminoglycosides or the noise-induced hearing loss type. In these cases, the toxic components are converted into hydroxyl radicals, causing the death of cochlear hair cells^3^. Nonetheless, in normal metabolic processes, important ORS (superoxide anions, hydrogen peroxide and hydroxyl radicals) are also generated during O_2_ and H_2_O reduction. Thus, enzymes belonging to the antioxidant system, which act on the metabolism of glutathione and on the decomposition of superoxide anions and hydrogen peroxide, are activated on an attempt to neutralize these molecules which potentially damage the cochlear tissue^4^.

Glutathione (GSH) detoxifies a number of exogenous and endogenous substances, by a non-enzymatic pathway and by enzymatic conjugation of the metabolites catalyzed by S-Transferase Glutathione (GST)^5^. GSTs are broken down into eight gene classes which codify cytosolic enzymes and microsomal genes: α (GSTA), μ (GSTM), ω (GSTP), θ (GSTT), z (GSTZ), α (GSTS), μ (GSTO) and k (GSTK)6. The GST enzymes have different affinities to numerous substrates and are found in many tissues at different concentrations^7^.

In humans, two of these subclasses GSTM1 and GSTT1, have genetic variability. About 30-50% of the individuals had a null genotype for gene GSTM1, depending on the race^8^ and 25-40% carried the null genotype of gene GSTT^19^. Individuals with the null genotype do not conjugate metabolites or specific toxins for these enzymes, thus increasing susceptibility to damage caused by oxidative stress and, possibly, the noise-induced hearing deficit^10^.

Changes to the antioxidant system and an increase in the oxidative stress can potentialize the toxicological responses associated with glutathione reduction. This indicates that the radicals generated modulate the activity and the expression of the antioxidant enzymes^11^. In the cochlea, the antioxidant activities play a crucial role on the reduction of damage caused by ototoxic agents, noise and aging. In vitro, gentamicin forms a complex with iron, producing free radicals^12^. Similarly, in vivo, it increases the levels of peroxide in the lipids^13^. In an external hair cell culture, glutathione prevents gentamicin-induced cytotoxicity and dampens the antibiotic-induced hearing loss in malnourished animals^14^. Thus, this study aimed at assessing the genetic variability of genes GSTM1 and GSTT1 and the ototoxic susceptibility of the aminoglycoside antibiotics in normal-hearing individuals and those with hearing impairment.

## MATERIALS AND METHODS

### Group selection

The study was carried out in individuals with hearing impairment (HI) who used or did not use aminoglycoside ototoxic antibiotics, and in hearing newborns, premature and high-risk babies who remained at least 48 hours in the neonatal ICU and were exposed to the antibiotic. The patients were broken down into three groups: group A, individuals with hearing impairment and who used amino-glycosides (n=10); group B, with hearing impairment and who did not use aminoglycosides (n=20); and group C, hearing individuals who used aminoglycosides (n=20). The control group was made up of 72 individuals, randomly selected from the general population.

### Molecular analysis of genes GSTM1 and GSTT1

Samples of genomic DNA were extracted from the peripheral blood after obtaining the signed consent form approved by the Ethics Committee (Approval # 484/2006). In order to extract the DNA from individuals from the general population we used the DNAzol reagent, according to instructions from the manufacturer (Invitrogen®). In the samples from the other groups we used the phenol-chloroform standard.

**Figure 1:**
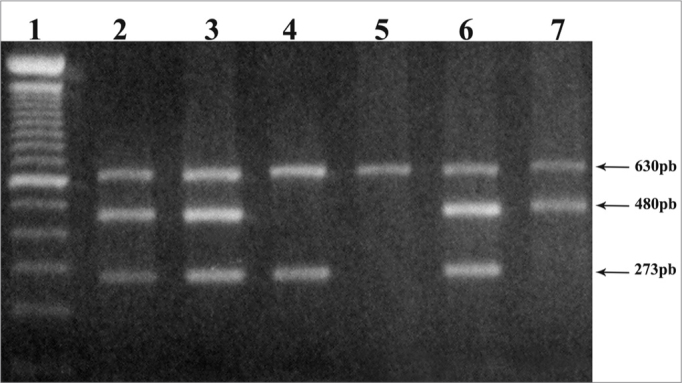
Null GSTM1 and GSTT1 genotype detection. Electrophoresis in 1.5% agarose gel showing the products of PCR-multiplex of 273bp and 480pb, correspondent. Presence of the normal allele of genes GSTM1 and GSTT1, respectively. The 630pb fragment represents the internal amplification control. (1) – molecular weight marker (100pb); (2,3,6) – individuals with normal GSTM1 and GSTT1 alleles, (4) – individual with a null allele for gene GSTT1, (5) – individuals with null combined allele for genes GSTM1 and GSTT1, (7) – individual with null allele for gene GSTM1.

The polymorphic sequences of genes GSTM1 and GSTT1 were established by a multiplex PCR, including the b-globin gene as internal control of the 630pb^15^ amplification. The reaction was carried out using 200 to 500ng of the genomic DNA, 10mM of solution with deoxynucleotides (dATP, dCTP, dGTP and dTTP), 20pmol/ml of the b-globin gene primers, 10pmol/ml of each primer from genes GST; 2.5U of Taq DNA polymerase; 10X PCR buffer (Tris-HCl 10mM pH 8.8) and 3mM of MgCl2, in a final volume of 50ml. PCR-multiplex conditions happened by an initial denaturation at 95°C for 3min followed by 35 cycles (95°C for 1min; 60°C per 1min; 72°C per 1min) and a final extension at 72°C for 10min. The PCR products were analyzed in 1.5% agarose gel dyed in ethide bromide, and the null genotype (both alleles deleted) for genes GSTT1 and GSTM1 was identified by the absence of amplification fragments of 480pb and 273pb, respectively (Fig.1).

### Statistical Analysis

The statistical significance differences between the groups were calculated by c2 followed by the Yates correction with a 5% significance level (p £ 0.05 values are considered significant).

## RESULTS

The genotypes were grouped by hearing normality, presence of hearing impairment and exposure to aminoglycoside antibiotics. The null GSTT1 was found in 16% (08 of 50) of the patients and in 18% (13 of 72) of the control individuals (p= 0.009). Null GSTM1 alleles were found in 21 (42%) of the 50 patients studied and in 38 (53%) individuals from the general population (p= 0.049). The frequency of homozygote GSTT1 and GSTM1 genotypes was significantly lower in patients assessed when compared to control individuals.

The frequency of null GSTT1 and GSTM1 genotypes and the comparison of the results among the groups are shown on Table 1. Nonetheless, no significant statistical difference was seen in groups control and A (p= 0.86) and (p= 0.41), control and B (p= 0.27) and (p= 0.24), control and C (p= 0.07) and (p= 0.47), control and A+C (p= 0.09) and (p= 0.47), C and A (p= 0.32) and (p= 0.75), GSTT1 and GSTM1, respectively.

**Table 1:** Null genotypes from genes GSTT1 and GSTM1 in control individuals and in patients with and without exposure to aminoglycosides.

Genotypes*	Group A (n = 10)	Group B (n=20)	Group C (n=20)	Control (n=72)
	n (%)	n (%)	n (%)	n (%)
GSTM1				
Positive [+]	6 (60)	12 (60)	11 (55)	34 (47)
Null [–]	4 (40)	8 (40)	9 (45)	38 (53)
GSTT1				
Positive [+]	8 (80)	17 (85)	13 (65)	59 (82)
Null [–]	2 (20)	3 (15)	7 (35)	13 (18)
Combined GSTM1/GSTT1				
[–]/[–]	2 (20)	0 (0)	4 (20)	6 (9)
[+]/[+]	6 (60)	9 (45)	8 (40)	27 (37)
[+]/[–]	0 (0)	3 (15)	3 (15)	7 (10)
[–]/[+]	2 (20)	8 (40)	5 (25)	32 (44)

[–]: null genotype; [+]: non-null genotype.

## DISCUSSION

Antioxidant and detoxifying reactions related to glutathione are essential for intracellular protection. Nonetheless, it is known that the treatment with some drugs and time affect the inner ear defense system of mammals. Noise exposure and treatment with gentamicin do not affect tissue glutathione levels. Nonetheless, the antioxidant system is being influenced by aging and by the use of cisplatin^16^.

Many studies suggest that the antioxidant activity can impact the hearing loss susceptibility. There are reports of genetic polymorphism on GSTM1 which act against the ototoxicity of cisplatin^17^, besides showing an apparent protective effect on the external hair cells in noise-exposed individuals^10^. In such a case, individuals with null genotype for the GSTM1 gene, on the otoacoustic emission, have lower amplitude for higher frequencies when compared to individuals without polymorphism in this gene. On the other hand, in another study, we did not show any evidence that the genetic variability of the antioxidant system prevails over other potential factors on the susceptibility regarding noise-induced hearing loss^18^.

The availability of information on GSTT1 and GSTM1 polymorphisms in the Brazilian population is still scarce^19^. A study carried out in the Brazilian population from the states of Pará and São Paulo showed frequencies of 18% and 47.3% for the null genotypes for genes GSTT1 and GSTM1, respectively^20^. In this paper, the frequencies found were very similar, null genotype for GSTT1 was of 16% and for GSTM1 it was 42%. Notwithstanding, there is no data in the literature for glutathione S-transferase (GSTT1 and GSTM1) polymorphism be associated to the susceptibility of aminoglycoside-induced hearing loss.

Nonetheless, we know that there are numerous environmental factors which can cause hearing loss, such as glutathione S-transferase that have an important role to play on the defense against oxidative stress caused by these factors.

## CONCLUSION

Our data strongly suggests that the null GSTT1 and GSTM1 genotypes are not associated to aminoglycoside-induced hearing loss.
